# Supplementary material for: The impact of the COVID-19 pandemic on oral anticoagulation adherence in patients with atrial fibrillation managed in primary care: Results from the PRIME Registry
Source: PLoS One. 2026 Mar 6;21(3):e0344020. doi: 10.1371/journal.pone.0344020 (PMC12965529; doi:10.1371/journal.pone.0344020)
Supplement: Attachment — Submitted filename: Response to Reviewers.pdf [file pone.0344020.s004.pdf]

November 9, 2025

Dear Editor and reviewers,

Thank you for the opportunity to resubmit our manuscript entitled, "The impact of the COVID-19 pandemic on oral anticoagulation adherence in patients with atrial fibrillation managed in primary care: Results from the PRIME Registry" to PLOS ONE.

We greatly appreciate the time and attention you have given to our manuscript. Below we outline the changes we have made in response to your comments. Page references in our responses refer to the page where the response/revision begins in the tracked version of the manuscript.

We found the comments and suggestions very helpful and feel our revised manuscript is much stronger. We hope we have sufficiently addressed all comments and concerns but are happy to make any additional changes if needed. Thank you for your time and consideration.
Sincerely,

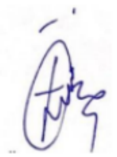

Omer Atac, MD, PhD
Department of Public Health, International School of Medicine
Istanbul Medipol University
Istanbul, Türkiye
+90 (554) 33554 56
oatac@medipol.edu.tr

ACADEMIC EDITOR:

1. **Clarify Pre-Pandemic Decline**

The sharp drop in persistence from 49.9% to 28.3% before the pandemic is unusually large. Provide additional context, sensitivity analysis, or discussion to explain this finding.

Response: Thank you for pointing this out. We agree that this drop is larger than typically reported in the literature, which estimates approximately 12–15% annual decline in warfarin users. We have revised the Discussion to offer a more robust interpretation of this trend. Specifically, we note that our sample included only patients with prior OAC prescriptions, which may enrich for individuals approaching discontinuation. We also discuss the possibility that prescriptions outside primary care settings (e.g., specialty clinics) were not captured in the EHR data, particularly in the Pre-1 year. Additionally, the smaller number of patients in later periods may have amplified percentage changes. Together, these factors may have contributed to the

steeper-than-expected drop and should be considered when interpreting the pandemic-related effects **(pages 15-16, lines 273-301)**.

2. **Expand Methods on Data Handling**

Clearly describe handling of missing data, exclusion criteria impact (e.g., how many patients excluded), and potential selection bias.

Response: Thank you for this helpful comment. We have expanded the Methods section to clarify the handling of missing data and the application of inclusion and exclusion criteria. We now note that the PRIME Registry includes a large national sample of clinical data over 6.5 million patients across 70 million visits from which our analytic sample was drawn. To ensure data quality and analytic integrity, we applied exclusion criteria in two stages:
(1) At the practice level, we excluded those with low volume (<250 visits per year) or prolonged service gaps (≥6 months), to ensure a consistent level of care delivery across the study period.
(2) At the patient level, we excluded cases with missing key demographic or clinical variables (e.g., age, sex, prescription dates), all of which were required for defining persistence and covariates. These exclusions occurred before the final cohort of 3,010 patients was constructed. As the proportion of missingness was very low (<1%), no imputation methods were applied.

We also acknowledged in the Limitations section that these steps, while necessary to ensure analytic rigor, may introduce selection bias by disproportionately excluding patients with lower engagement or less complete records **(page 7, lines 106-121; pages 19-20, lines 361-392)**.

3. **Refine Interrupted Time Series (ITS) Analysis**

Report confidence intervals for ITS coefficients, check for autocorrelation, and justify model choice (segmented regression vs alternatives).

Response: We appreciate the reviewer's suggestion to improve the methodological transparency of our Interrupted Time Series (ITS) analysis. Below, we provide additional clarification and robustness checks in response to the specific points raised.

1. Reporting Confidence Intervals

We now report 95% confidence intervals (CIs) for all ITS model coefficients in the Results section and in Table 3. This provides readers with a clearer understanding of the precision of the effect estimates for both level and trend changes across study periods.

2. Checking for Autocorrelation

We have tested for autocorrelation in the residuals of the ITS model using the Durbin-Watson statistic and did not indicate significant autocorrelation (Durbin-Watson = 2.14, close to the ideal value of 2.0), suggesting that autocorrelation is unlikely to bias our estimates. We have added this information to the Methods section under the ITS subsection and included a brief note in the Results section to affirm the robustness of the model.

3. Justification for Segmented Regression

We chose segmented linear regression as our primary ITS model due to the quarterly nature of our data, the relatively limited number of time points (n = 20), and the clear a priori break point (start of the COVID-19 pandemic). Segmented regression is well-suited for evaluating changes in both level and trend across defined periods in observational data and is widely used in healthcare services research for this purpose [1, 2]. We have now expanded the justification in the Methods section and cited relevant literature supporting the suitability of segmented regression in this context.

References

1. Penfold RB, Zhang F. Use of interrupted time series analysis in evaluating health care quality improvements. Acad Pediatr. 2013;13(6):S38–S44. doi:10.1016/j.acap.2013.08.002

2. Lopez Bernal J, Cummins S, Gasparrini A. Interrupted time series regression for the evaluation of public health interventions: a tutorial. Int J Epidemiol. 2017;46(1):348–355. doi:10.1093/ije/dyw098

4. **Improve Adherence Definition & Threshold Justification**

Discuss rationale for using ≥80% as “good adherence” and interpret findings using higher thresholds (≥95%) given clinical relevance.

Response: We thank the editor for this thoughtful comment. We agree that further clarification was warranted regarding the rationale for using the ≥80% cutoff and the clinical interpretation of higher adherence thresholds. The ≥80% value was selected because it is the conventional definition of “good adherence” in drug utilization adherence research, allowing comparability with prior studies and meta-analytic standards. However, we recognize that this threshold is empirical and may underestimate clinically relevant nonadherence for oral anticoagulants. We have therefore expanded the discussion to explain this rationale and to explicitly address higher adherence thresholds (≥90–95%), emphasizing its consequences. The revised paragraph now interprets our findings in light of both methodological convention and clinical relevance, highlighting the importance of using stricter thresholds for OAC adherence assessment as such:

“The conventional adherence threshold of ≥80% widely used in drug utilization research was applied in our study to ensure comparability with prior literature and meta-analytic standards [29]. However, this cutoff is largely empirical and may underestimate clinically meaningful nonadherence for oral anticoagulants. This level of poor adherence likely has devastating consequences, Even brief discontinuation periods as short as one week have been associated with mortality, regardless of the OAC used [30], underscoring the need for more stringent adherence benchmarks.. Because of the short half-lives and narrow therapeutic windows of these agents, several recent studies have advocated adopting higher thresholds (≥90–95%) to better capture clinically optimal adherence [4, 31]. **(page 17, lines 312-324)**.

5. **Explore Subgroup Differences**

Conduct additional subgroup analysis (e.g., by drug class, age, sex, comorbidity level) to better explain heterogeneity in adherence trends.

Response: We appreciate the suggestion to conduct subgroup analyses to explore heterogeneity in adherence and persistence trends. We emphasize that our study already includes detailed multivariable logistic regression models that examine persistence and adherence across key subgroups, including drug class (NOAC vs. VKA), age, sex, comorbidity level (CHA_2DS_2–VASc and CCI), rurality, and social deprivation index; presented in Figure 3a and 3b, and the corresponding results are described in the text.

Our findings indicate notable differences across subgroups. For instance, NOAC users had significantly lower odds of persistence in pre-pandemic years but higher odds in PY-2. Rural patients and those residing in high-SDI areas were more likely to remain persistent and adherent during the pandemic. These variations highlight important contextual and structural factors affecting medication use and support the need for continued subgroup-focused inquiry. We have clarified this point further in the Discussion section **(pages 17-19, lines 325-360)**.

6. **Address Rural vs Urban Paradox**

Provide more interpretation or potential mechanisms for why rural patients showed higher persistence and adherence during the pandemic.

Response: Thank you for these important observations. We have revised the Discussion section to expand on potential explanations for the better adherence and persistence outcomes among rural and high-SDI patients. Specifically, we now cite prior evidence suggesting that rural populations may exhibit stronger patient-provider continuity, more stable care-seeking behaviors, or greater reliance on primary care providers during public health disruptions. We also acknowledge that our findings may partly reflect sample composition in the later study periods, when total patient numbers declined potentially influencing subgroup patterns. To address these concerns, we have softened speculative interpretations and clearly stated that additional research is needed to confirm mechanisms behind these findings **(pages 18-19, lines 348-360)**.

7. **Strengthen Limitations Section**

Emphasize limitations such as lack of pharmacy claims linkage, possible overestimation of discontinuation, and generalizability outside U.S. primary care.

Response: Thank you for the suggestion to elaborate on the study limitations. We have revised the Limitations section to better acknowledge key constraints of our data and design. Specifically, we now address the absence of pharmacy claims linkage and its implications for estimating discontinuation or adherence. We also added points on potential selection bias introduced through our exclusion criteria, and clarified the limitations in generalizability beyond U.S. primary care settings **(pages 19-20, lines 361-392)**.

8. **Improve Figures & Tables for Clarity**

Add denominators to tables, include confidence intervals in Figure 3 logistic regression plots, and ensure all figures are fully self-explanatory.

Response: Thank you for the suggestion. We have now added exact denominators (n) for each study period in the rows for both persistence and adherence subgroups, calculated using the full sample (n = 3,010) and period-specific persistence rates.

We also appreciate the reviewer's suggestions to improve the clarity of Figure 3a and 3b. In response:

- Adjusted Odds Ratios with 95% Confidence Intervals were already included and visually displayed in both figures. To make this explicit, we added the phrase "Adjusted Odds Ratios 95% CI" as a subtitle within the figure panels.

- We have retained the current format to ensure the figures remain self-explanatory and aligned with the journal's visual guidelines.

We hope these clarifications and minor adjustments address the concerns raised.

Review Comments to the Author

Reviewer #1:

This is a clinically relevant study addressing the impact of the COVID-19 pandemic on oral anticoagulation adherence and persistence in atrial fibrillation patients in primary care. The study uses a large, nationwide dataset (PRIME registry) and applies robust statistical methods (interrupted time series and logistic regression).

Comment 1: Abstract: The results section is slightly overloaded; consider condensing while keeping key findings (persistence decline, adherence decline, ITS result).

Response 1: Thank you for the suggestion. We have revised the Results section of the Abstract to improve clarity and conciseness while retaining the key findings on persistence, adherence, the ITS analysis, and the pattern of NOAC use **(page 2, lines 38-43)**.

Comment 2: Keywords: Consider adding "warfarin" and "NOAC" as keywords for indexing.

Response 2: Thank you for the comment. We have now added "warfarin" and "NOAC" to the list of keywords to enhance indexing.

Comment 3: Figures: Figures 2 and 3 should include clearer legends with sample sizes and clearer y-axis labels.

Response: Thank you for the suggestion. We appreciate your emphasis on clarity. Upon careful review, we believe that the current version of Figure 2 already includes sufficient labeling, with a clearly defined y-axis (persistence rate in percentage), an x-axis indicating quarterly time points, and a red dashed line denoting the start of the pandemic intervention. The sample size (n =

3,010) is now also included in the updated title of Table 2, which directly informs the figure. As such, we did not modify Figure 2 further, but we remain open to additional revisions if the editor recommends any changes.

We also appreciated the reviewer's suggestions to improve the clarity of Figure 3a and 3b. In response:

- Adjusted Odds Ratios with 95% Confidence Intervals were already included and visually displayed in both figures. To make this explicit, we added the phrase "Adjusted Odds Ratios with 95% CI" as a subtitle within the figure panels.
- Category labels, reference groups, and study periods were already indicated clearly in the current version.
- Y-axis labels are consistent across both figures, and decimal formatting was standardized.
- We have retained the current format to ensure the figures remain self-explanatory and aligned with the journal's visual guidelines.

We hope these clarifications and minor adjustments address the concerns raised.

Comment 4: Ethics: The manuscript states no consent was obtained due to de-identified data, which is fine, but it would be helpful to explicitly state that this complies with US regulations for secondary EHR research.

Response 4: We appreciate the reviewer's suggestion. We have now clarified in the Ethics section that the waiver of informed consent complies with U.S. federal regulations for secondary analysis of de-identified EHR data **(page 10, lines 194-198)**.

Comment 5: The description of patient inclusion is somewhat confusing. It would help to clarify why only patients with ≥2 OAC prescriptions in the pre-study period (2016–2017) were included and how this may affect generalizability.

Response 5: We appreciate the reviewer's observation. We have clarified the rationale for requiring ≥2 OAC prescriptions during the pre-study period. This criterion was used to ensure the inclusion of patients who had been on treatment for a sustained period prior to baseline, allowing us to more reliably observe longitudinal patterns in medication-taking behavior. The revised explanation has been added to the Methods section under "Study Population" **(page 7, lines 103-121)**.

Comment 6: The rationale for excluding patients from practices with <250 visits/year or gaps in service should be expanded—does this exclusion risk introducing selection bias?

Response 6: Thank you for this helpful comment. We clarified in the Methods section that the exclusion of practices with low visit counts or prolonged service gaps aimed to ensure that only clinics consistently providing care were included **(page 7, lines 103-121)**.

Comment 7: Persistence is defined using the treatment anniversary method with a 1-month gap, but this may underestimate persistence compared to other definitions. A justification for this choice should be provided, ideally with a sensitivity analysis.

Response: We acknowledge that the 1-month grace period in the treatment anniversary method may yield more conservative persistence estimates than other methods using longer permissible gaps (e.g., 60 or 90 days). However, we intentionally selected this stricter definition to capture meaningful gaps in OAC therapy, particularly given the high-risk profile of the atrial fibrillation population in our study. Even short discontinuations can lead to serious clinical consequences [Ref 30], especially among patients using warfarin, which comprised the majority of our sample. This method offers a standardized and clinically meaningful definition of persistence, balancing overestimation (by allowing indefinite gaps) and underestimation (by using too narrow a window). As noted in the limitations section, we acknowledge that this definition may underestimate persistence in some cases, especially if prescriptions were filled outside the primary care setting or if continuity was maintained through other healthcare channels. However, alternative definitions would also be subject to misclassification given the structure of our EHR-based dataset **(pages 19-20, lines 361-392)**.

Comment 8: Adherence was only measured in persistent patients. This approach excludes early discontinuers and may bias results. A sensitivity analysis including all patients would strengthen the conclusions.
Response: Thank you for this insightful comment. As noted, our adherence analyses were limited to patients classified as persistent in each study period. This approach was chosen to focus specifically on compliance behavior among those who remained on therapy, consistent with prior studies. However, we agree that excluding early discontinuers may lead to overestimation of overall adherence **(pages 19-20, lines 361-392)**.

Comment 9: The interrupted time series (ITS) results are only partially explained. The manuscript notes a significant immediate drop in persistence but not in trends. A clearer interpretation of the coefficients and effect sizes is needed.

Response: We thank the reviewer for highlighting the need for a more explicit interpretation of the ITS results. In the revised manuscript, we have expanded our results and discussion of the ITS coefficients to clarify the magnitude and statistical significance of the observed changes **(pages 13, lines 234-239; pages 15-16, lines 265-301)**.

Comment 10: The logistic regression models yield very wide confidence intervals (e.g., NOAC OR = 12.50, 95% CI: 1.32–100.00), suggesting instability due to small subgroup sizes. The authors should acknowledge this limitation more explicitly.

Response 10: We appreciate your attention to the width of the confidence intervals in some of our logistic regression results. As you rightly note, the wide interval likely reflects the smaller sample sizes for certain subgroups, especially during the pandemic period. We have explicitly acknowledged this issue in the Limitations section and clarified that these results should be interpreted with caution due to potential statistical instability stemming from small cell sizes **(pages 19-20, lines 361-392)**.

Comment 11: The decline in persistence is extremely steep (49.9% → 28.3% → 8.2%). This appears more dramatic than in previous literature. The authors note this but should further explore possible data quality issues or biases in EHR records (e.g., unrecorded prescriptions from outside primary care).

Response: Thank you for this important comment. We agree that the observed decline in persistence from 49.9% to 8.2% is steeper than what has been previously reported in the literature. While our analyses showed consistent trends across time, we have now expanded our discussion of potential data limitations that could contribute to this sharp drop. Specifically, we have added a paragraph noting the possibility that prescriptions filled outside of the primary care setting (e.g., cardiology or urgent care) may not have been captured in the EHR data. This could result in an underestimation of persistence, particularly during the pandemic when patients may have sought care through alternative routes. Additionally, we highlight the decreasing number of patients in later time periods, which may reflect both true attrition and reduced data completeness. These points are now explicitly discussed in the limitations section **(pages 19-20, lines 361-392)**.

Comment 12: Tables and figures could be improved for clarity. For example, Table 2 might be simplified to highlight key adherence thresholds (≥95%, ≥80%) instead of multiple small subcategories.

Response: We appreciate the suggestions to improve the clarity of Table 2. In response to the suggestion to simplify the table, we retained the detailed adherence subgroups because they help show the gradient within the "Good adherence" category, especially the decline in highest adherence (≥95%). However, to make the table more accessible, we highlighted key adherence thresholds (≥80% and ≥95%) in the text. To avoid any confusion, we have revised the table layout to clearly group these rows under the "Good adherence" and have added a footnote to clarify this. We have also explicitly stated the total sample size (n = 3,010) in the table title and number of participants in each group.

Comment 13: The discussion cites contrasting findings (e.g., improved adherence in claims-based studies). The manuscript should elaborate on why results differ (primary care vs cardiology care, registry vs claims data).

Response: We thank the reviewer for this valuable comment and agree that additional clarification was needed to explain why our findings differ from those of claims-based studies. We have expanded this section of the *Discussion* to elaborate on key methodological and contextual differences, including data source, care setting, patient population. The revised paragraph now clarifies that these factors likely explain the apparent discrepancy between studies and emphasizes that differences across data environments reflect complementary perspectives on adherence behavior rather than contradictions as such:
"At first glance, these results appear inconsistent with ours, but the difference largely reflects variation in data source, care context, and study population: Claims data primarily capture dispensing and insurance transactions that measure medication possession rather than confirmed use, often encompassing prescriptions from cardiology, specialty, and hospital settings. In contrast, our primary-care EHR data capture longitudinal prescribing activity within community practices, offering a closer view of ongoing therapy management and care disruptions. Moreover, registry-based EHR cohorts such as ours typically include established, older users, whereas claims databases often represent mixed or newly treated populations. Thus, while claims data may show short-term refill surges or stockpiling early in the pandemic, practice-based EHR data reveal longer-term discontinuation and reduced treatment continuity. These complementary perspectives highlight how adherence and persistence trends can differ depending on data environment and clinical setting, reinforcing the need for contextual interpretation when comparing studies." **(pages 16, lines 288-301)**.

Comment 14: The explanation for better adherence among rural and high-SDI patients is interesting but speculative. Additional references or supporting analyses would strengthen this point.

Response: Thank you for these important observations. We have revised the Discussion section to expand on potential explanations for the better adherence and persistence outcomes among rural and high-SDI patients. Specifically, we now cite prior evidence suggesting that rural populations may exhibit stronger patient-provider continuity, more stable care-seeking behaviors, or greater reliance on primary care providers during public health disruptions. We also acknowledge that our findings may partly reflect sample composition in the later study periods, when total patient numbers declined potentially influencing subgroup patterns. To address these concerns, we have softened speculative interpretations and clearly stated that additional research is needed to confirm mechanisms behind these findings **(pages 18-19, lines 348-360)**.

Comment 15: The study's limitations section is appropriate but could be expanded to emphasize:
Lack of pharmacy claims linkage (risk of misclassification).
Overrepresentation of warfarin (93%)—limiting generalizability to NOAC users.
Predominantly white sample (89%)—limiting applicability to more diverse populations.

Response 15: Thank you for the valuable suggestions. We expanded the Limitations section to explicitly address the lack of pharmacy claims linkage, the predominance of warfarin users in our sample, and the limited racial/ethnic diversity **(pages 19-20, lines 361-392)**.

Comment 16: Some sentences are lengthy and complex. Shortening and simplifying would improve readability. For example, the paragraph describing the pre-pandemic decline could be condensed.

Response 16: Thank you for this suggestion. We carefully reviewed the manuscript and revised several long or complex sentences for clarity and readability.

Comment 17: Occasional tense inconsistencies (past vs present) should be corrected.

Response 17: We appreciate this helpful observation. We reviewed the manuscript and corrected inconsistencies in tense usage. All methods and results are now presented in past tense for consistency, while background and general knowledge statements remain in present tense where appropriate.

Comment 18: The following reference might be helpful.
https://doi.org/10.1142/S2737416525500772

Response 18: We reviewed the suggested reference but could not identify any findings relevant to our focus on warfarin vs NOAC persistence, COVID-19 impacts, or primary care settings, so we did not include it in our analyses.

Reviewer #2:

Comment 1: The sharp decline in persistence from 49.9% (Pre-2) to 28.3% (Pre-1) before the pandemic is striking and requires more detailed discussion. The authors attribute this to expected annual decline and "significant challenges", however, the reported drop is nearly double what is expected from other literature. The manuscript needs a more robust discussion or analysis to explain this pre-pandemic trend, as it significantly impacts the interpretation of the pandemic's effect.

Response: Thank you for pointing this out. We agree that this drop is larger than typically reported in the literature, which estimates approximately 12–15% annual decline in warfarin users. We have revised the Discussion to offer a more robust interpretation of this trend. Specifically, we note that our sample included only patients with prior OAC prescriptions, which may enrich for individuals approaching discontinuation. We also discuss the possibility that prescriptions outside primary care settings (e.g., specialty clinics) were not captured in the EHR data, particularly in the Pre-1 year. Additionally, the smaller number of patients in later periods may have amplified percentage changes. Together, these factors may have contributed to the

steeper-than-expected drop and should be considered when interpreting the pandemic-related effects **(pages 15-17, lines 265-311)**.

Comment 2: It is mentioned that; "significant immediate level change in the first quarter of the pandemic (coefficient: -24.99, p=0.001)". However, the accompanying Figure 2 shows quarterly rates that are all well below 70%, with the pre-pandemic trend appearing to be around 72%, and the pandemic-period trend line starting at around 58%. The persistence rates in Table 2 are annual and much lower, for example, 8.2% in PY-1. This discrepancy between the quantitative results in the text, the figure, and the table is highly confusing and needs to be clarified.

Response: We appreciate this comment and fully agree that clarification was needed. To address this, we have now revised both the Results section and the titles of Table 2 to make clear that the Table 2 presents annual persistence using the treatment anniversary method. These are distinct operationalizations of persistence: the former provides higher temporal resolution based on quarterly counts, while the latter offers a conservative estimate of full-year persistence with a 30-day permissible gap.

Additionally, we have clarified in the Results text that the ITS analysis captures a sharp level drop in persistence between the last pre-pandemic quarter and the first pandemic quarter (Q2 2020), which is visualized in Figure 2 **(pages 13, lines 234-239)**.

We hope this improved clarity resolves the confusion noted, and we thank the reviewer again for highlighting this issue.

Comment 3:
The manuscript's conclusion that the pandemic caused a sustained decline in adherence and persistence appears to be contradicted by some of the adherence data. While persistence remains low, the "Good adherence, %" and "80-84" to "90-94" adherence rates show an upward trend from PY-1 to Post-1. The authors need to reconcile this in their discussion.

Response: We thank the reviewer for this thoughtful observation. We agree that certain adherence categories (for instance, 80–94%) show a modest upward trend between PY-1 and Post-1. However, we would like to clarify that adherence was calculated only for patients who remained persistent during each observation period, as defined by the treatment anniversary method. Because overall persistence declined sharply over time, the number of patients contributing to the adherence calculations became progressively smaller and more selective largely consisting of individuals with inherently higher adherence.

The apparent improvement in adherence within these subgroups therefore reflects selective retention (survivor bias) among persistent patients, rather than a genuine recovery of adherence at the population level. To make this distinction clearer, we have added the numerator and denominator counts to Table 2. This allows readers to see how the persistent cohort contracts over time and to interpret the conditional adherence percentages more accurately in that context.

The primary outcome persistence still shows a marked and sustained decline across the study period, reinforcing our conclusion that the pandemic had a lasting negative impact on continuity of anticoagulation therapy. We have now revised the Discussion section to make this denominator effect and the difference between persistence and adherence among persistent users explicitly clear as such:

"This trend partly arises because adherence was evaluated only among patients who remained persistent under the anniversary-based definition; as overall persistence declined, the remaining cohort represented a progressively smaller and inherently more adherent group. Consequently, modest increases observed in some intermediate adherence categories (e.g., 80–94%) reflect the selective retention of highly adherent patients rather than a true improvement in adherence behavior. This persistence-conditioned pattern underscores that, despite apparently stable adherence within this subgroup, overall treatment continuity continued to deteriorate at the population level." **(pages 16-17, lines 302-311)**.

Reviewer #3:

Abstract

Comment 1: The finding for NOACs is unclear ("higher during the pandemic year-2" - higher what?).

Response 1: Thank you for pointing this out. We have clarified the sentence in the abstract to indicate that the "higher" refers to higher persistence among NOAC users during pandemic year-2 relative to warfarin users **(page 2, lines 42-43)**.

Comment 2: The chosen pre-pandemic persistence comparison (2018-19 vs. pandemic year-1) is misleading; the immediate pre-pandemic year (2019-20) is more relevant.

Response 2: Thank you for this observation. We understand the concern; however, our time periods were defined from March 15 to March 14 in each year to align with the onset of the COVID-19 pandemic in the United States (March 15, 2019). Therefore, what we referred to as the "pre-pandemic year" is March 15, 2018, to March 14, 2019, and the "pandemic year-1" is March 15, 2019, to March 14, 2020. While the 2019–2020 period may appear to be the immediate pre-pandemic year, it partially overlaps with the early pandemic phase and was not used as a baseline comparison to avoid contamination.

Comment 3: The persistence drop is stated as "from 49.9% in 2018–2019 to 8.2% in the pandemic year-1". However, "2018-2019" is the Pre-2 period, but the most relevant pre-pandemic comparison is the immediate pre-pandemic year (Pre-1: 28.3%). The chosen comparison exaggerates the drop. The comparison to Pre-1 (28.3% to 8.2%) is still stark and more accurate.

Response 3: Thank you for this thoughtful observation. We appreciate your concern about the comparison period. To clarify, our study defines each year from March 15 to March 14 of the following calendar year to align with the onset of the COVID-19 pandemic on March 15, 2019. As such:

Pre-pandemic year (Pre-1): March 15, 2018 – March 14, 2019
Pandemic year-1: March 15, 2019 – March 14, 2020

We intentionally used 2018–2019 (Pre-1) as our comparison period to avoid contamination from early pandemic disruptions.

Methods

Comment 4: Incorrect study design classification; this is a cohort study, not a cross-sectional study.

Response 4: Thank you for pointing this out. We agree with your assessment. While the original version of the manuscript described the study as cross-sectional, the design is more accurately characterized as a retrospective cohort study, as we followed a defined population over time to assess persistence and adherence patterns **(page 6, line 80)**.

Comment 5: The "Post-1" period (March 2022-2023) is mislabeled as "post-pandemic"; it was still during the pandemic.

Response 5: Thank you for this important observation. We acknowledge that the term "post-pandemic" may not universally reflect the reality of the ongoing COVID-19 situation during the 2022–2023 period. Our intention was to differentiate the initial pandemic years characterized by acute disruption from the more stabilized, late-pandemic period in terms of primary care operations and medication use patterns.

Comment 6: Calculating adherence (PDC) only among persistent patients is a major methodological flaw that inflates adherence rates and limits comparability.

Response: We thank the reviewer for this important comment. We agree that calculating adherence (PDC) only among persistent patients can yield higher estimates compared to unconditional approaches. However, this was an intentional methodological choice, consistent with the conceptual distinction between adherence (day-to-day medication taking behavior) and persistence (continuation of therapy over a certain point or period of time). As described in Elseviers et al., *Drug Utilization Research: Methods and Applications*, these constructs are complementary but distinct and are ideally analyzed separately to avoid conflation. Our aim was to capture medication-taking behavior conditional on continued therapy, providing insight into how consistently patients maintained dosing among those who had not yet discontinued. This approach minimizes the downward bias in PDC that occurs when early discontinuers are

included in the denominator. Yet we appreciate your observation about the underlying assumptions and potential drawbacks of adherence measurement, which may not always be evident to readers less familiar with its methodological nuances. We have therefore clarified in the revised manuscript that, as with any adherence metric, calculating PDC among persistent patients involves inherent trade-offs between conceptual precision and cross-study comparability. To enhance transparency, this rationale is now elaborated in the *Methods* section and the limitation of conditional adherence is explicitly acknowledged in the *Limitations* section as such (for the latter):

"Adherence was calculated only among patients classified as persistent, an approach that enhances conceptual clarity by separating medication-taking behavior from treatment continuation but may also exclude early discontinuers, slightly overestimate adherence, and limit comparability with studies using unconditional definitions." **(pages 7-9, lines 124-163**; **pages 19-20, lines 361-392)**.

Results

Comment 7: Table 2 is confusing; the sub-categories of adherence do not clearly sum to the "Good adherence" total.

Response: We appreciate the suggestions to improve the clarity of Table 2. First, we would like to clarify that the subcategories listed under "Good adherence" (i.e., 80–84%, 85–89%, 90–94%, and 95–100%) do sum to the total "Good adherence" rate (≥80%) for each study period. To avoid any confusion, we have revised the table layout to clearly group these rows under the "Good adherence" and have added a footnote to clarify this. We have also explicitly stated the total sample size (n = 3,010) in the table title and number of participants in each group.

Comment 8: The results state "Adherence rates increased to 50.8% in PY-2 and 57.0% in Post-1". Given the drastic drop in persistence (to 4.1% and 2.6% in these periods), this "increase" is based on a tiny, potentially highly selected subset of patients.

Response: Thank you for this observation. We agree that the adherence rates reported for PY-2 and Post-1 should be interpreted cautiously due to the very low persistence rates in these periods (4.1% and 2.6%, respectively). To address this concern, we have made two key revisions: In the Results section, we now explicitly note that these adherence rates are based on a small, likely non-representative subgroup of patients who remained persistent. In the Discussion, we expanded the limitations **(page 12, lines 216-218; pages 19-20, lines 361-392)**.

Comment 9: Reporting an "increase" in adherence in later pandemic years (PY-2, Post-1) is misleading without the context of a catastrophically low persistence rate (<5%), making the sample size tiny and unrepresentative.

Response: Thank you for this observation. We agree that the adherence rates reported for PY-2 and Post-1 should be interpreted cautiously due to the very low persistence rates in these periods (4.1% and 2.6%, respectively). To address this concern, we have made two key revisions: In the Results section, we now explicitly note that these adherence rates are based on a small, likely non-representative subgroup of patients who remained persistent. In the Discussion, we expanded the limitations **(page 12, lines 216-218; pages 19-20, lines 361-392)**.

Discussion

Comment 10: The explanation for the contrast with Hernandez et al. is convoluted and unclear.
Response 10: Thank you for this helpful observation. We agree that our initial explanation was unclear. We have now revised the discussion to more clearly contrast our findings with those of Hernandez et al., emphasizing the key methodological and population differences (data source, inclusion criteria, and measurement definitions) that likely explain the divergent results **(page 16, lines 287-288)**.

Comment 11: The rationale for why rural/high SDI patients had better outcomes is speculative and unsupported by data.

Response: Thank you for these important observations. We have revised the Discussion section to expand on potential explanations for the better adherence and persistence outcomes among rural and high-SDI patients. Specifically, we now cite prior evidence suggesting that rural populations may exhibit stronger patient-provider continuity, more stable care-seeking behaviors, or greater reliance on primary care providers during public health disruptions. We also acknowledge that our findings may partly reflect sample composition in the later study periods, when total patient numbers declined potentially influencing subgroup patterns. To address these concerns, we have softened speculative interpretations and clearly stated that additional research is needed to confirm mechanisms behind these findings **(pages 18-19, lines 348-360)**.

Conclusions

Comment 12: Major contradiction; text recommends interventions for "low social deprivation areas," but results show "high" SDI was associated with better outcomes.

Response: We appreciate this careful reading. We have revised the conclusion to reflect our actual findings and correct the contradictory statement. The revised text now emphasizes that urban patients and those from socioeconomically advantaged backgrounds were associated with lower medication persistence and adherence during the pandemic, and therefore may benefit most from targeted support **(pages 20-21, lines 394-406)**.

Figures

Comment 13: Figures (Fig 2, 3a, 3b) are cited in the text but were not provided for review.

Response: Thank you for pointing this out. The figures (Fig 2, 3a, and 3b) have now been added to the revised manuscript and supplementary materials. We have also ensured that each figure is clearly referenced and placed accordingly in the text.
