# Supplementary material for: The impact of the COVID-19 pandemic on oral anticoagulation adherence in patients with atrial fibrillation managed in primary care: Results from the PRIME Registry
Source: PLoS One. 2026 Mar 6;21(3):e0344020. doi: 10.1371/journal.pone.0344020 (PMC12965529; doi:10.1371/journal.pone.0344020)
Supplement: Attachment — Submitted filename: Response_to_Reviewers_auresp_2.pdf [file pone.0344020.s005.pdf]

December 27, 2025

Dear Editor and reviewers,

Thank you for the opportunity to resubmit our manuscript entitled, "The impact of the COVID-19 pandemic on oral anticoagulation adherence in patients with atrial fibrillation managed in primary care: Results from the PRIME Registry" to PLOS ONE.

We greatly appreciate the time and attention you have given to our manuscript. Below we outline the changes we have made in response to your comments. Page references in our responses refer to the page where the response/revision begins in the tracked version of the manuscript.

We found the comments and suggestions very helpful and feel our revised manuscript is much stronger. We hope we have sufficiently addressed all comments and concerns but are happy to make any additional changes if needed. Thank you for your time and consideration.
Sincerely,

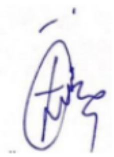

Omer Atac, MD, PhD
Institute of Population and Social Research
Marmara University
Istanbul, Türkiye
+90 (554) 33554 56
omer.atac@marmara.edu.tr

Reviewer #3:

Comment 1: Line 41-42 (Abstract): "NOAC use was associated with lower persistence pre-pandemic-, but with higher persistence during the pandemic year-2 compared to warfarin, but higher during the pandemic year-2." – This sentence is garbled, contains a stray hyphen and comma, and is repetitive.

Response: Thank you for pointing this out. We revised the sentence in the abstract to eliminate the stray punctuation, repetition, and improve clarity **(page 2, lines 42-44)**.

Comment 2: Line 49 (Keywords): "Warfarin, NOAC" – Inconsistent formatting; other keywords are separated by semicolons.

Response: Thank you for the comment. We have revised the keywords to ensure consistent use of semicolons throughout the list **(page 3, line 50)**.

Comment 3: Line 80 (Methods): "retrospective cohort cross-sectional study" – Appears to be a tracked-changes error, showing both "cohort" and "cross-sectional".

Response: Thank you for pointing this out. This was a tracked-changes typo. We have corrected this **(page 6, line 80)**.

Comment 4: Line 128 (Methods): "A patient was considered persistent if she has an active prescription..." – Tense inconsistency ("was considered" vs. "has"). Also, "she" is gendered language not used elsewhere.

Response: Thank you for pointing this out. We revised the sentence for consistency in both tense and language. Specifically, we replaced the gendered term “she” with the gender-neutral “they” and corrected the verb tense to maintain consistency within the sentence **(page 8, lines 126-127)**.

Comment 5: Line 135-136 (Methods): "avoids the underestimation that can occur when early discontinuers who by definition have zero coverage after discontinuation are included in the denominator to avoid potential confounding of early discontinuer compliance [17]," – Incomplete sentence and redundant phrasing due to tracked changes.

Response: We appreciate the reviewer pointing out the error introduced by tracked changes. We have revised the sentence to improve clarity and remove redundancy **(page 8, lines 132-134)**.

Comment 6: Line 199-200 (Methods, Ethics): "as outlined under the Department of Health and Human Services (HHS) guidelines (45 CFR 46.104, category 4)." – Sentence fragment.

Response: We appreciate the reviewer’s careful reading. The sentence in question is not a fragment, but rather a complete statement providing regulatory justification for the waiver of informed consent. As it currently reads clearly and accurately, no revision was made.

Comment 7: Line 214-217 (Results): "Persistence rates were highest during the Pre-2 period at 49.9% and lower declined in the following subsequent periods:" – Contains editing errors ("lower declined") and redundant words ("following subsequent").

Response: Thank you for the suggestion. We carefully reviewed the sentence, and it currently reads: "Persistence rates were highest during the Pre-2 period at 49.9%, and declined in the following periods: 28.3% in Pre-1, 8.2% in PY-1, 4.1% in PY-2, and 2.6% in Post-1 (Table 2)." We believe this version does not include the phrasing issues ("lower declined" or "following subsequent") mentioned in the comment. As such, no further revision was necessary.

Comment 8: Line 218-220 (Results): "Adherence rates also had a general downward trend, starting at 65.1% in Pre-2 and 64.1% in Pre-1 and falling to 48.6% in PY-1. Adherence rates–These rates reflect annual treatment anniversary persistence," – First sentence is incomplete. The dash after "rates" creates a fragment.

Response: Thank you for your close reading. We revised the sentence structure to improve clarity and remove any ambiguity or fragmentation. Specifically, we removed the redundant phrase "Adherence rates–These rates…" and reorganized the paragraph to clarify that persistence and adherence are distinct but related measures, and to ensure definitions are placed in the appropriate context **(page 12, lines 211-221)**.

Comment 9: Line 221-223 (Results): "Adherence rates, calculated among persistent patients only, also declined overall. They started at 65.1% in Pre-2 and 64.1% in Pre-1, then dropped to 48.6% in PY-1. Adherence rates appeared to increase to 50.8% in PY-2 and 57.0% in post-1 though rates remained below pre-pandemic levels;" – This repeats the adherence rates from the previous, now-incomplete sentence (Lines 218-220), creating redundancy and confusion.

Response: Thank you for pointing this out. To reduce redundancy and improve narrative clarity, we revised the structure and flow of the paragraph so that adherence rates are presented only once, in a logically sequenced and non-repetitive way **(page 12, lines 211-221)**.

Comment 10: Line 224 (Results): "these estimates are based on a very small subset of persistent patients (4.1% and 2.6% of the sample, respectively), increased to 50.8% in PY-2 and 57.0% in post-1." – This is a run-on sentence that incorrectly appends a repeated fact to a parenthetical clause.

Response: Thank you for catching this error in sentence structure and logic. The original sentence conflated two separate ideas, sample size and adherence rate trends, resulting in a confusing run-on structure. We revised the sentence to separate these points and ensure clarity **(page 12, lines 211-221)**.

Comment 11: Line 225 (Results): "Poor adherence rates were most prevalent..." – Previously, the term used is "poor adherence" (noun), not "poor adherence rates".

Response: We appreciate the reviewer's attention to detail. However, this concern appears to be based on an earlier draft. In the current version of the manuscript, we consistently use the term "poor adherence" (noun form), and do not refer to "poor adherence rates."

Comment 12: Line 227 (Results): "The proportion of patients with high adherence (MPR >95%) ..." – Inconsistent metric; the study uses PDC, not MPR (Medication Possession Ratio).

Response: We appreciate the reviewer's attention to this detail. We acknowledge the inconsistency in terminology. The study indeed uses Proportion of Days Covered (PDC) as the measure of adherence throughout. We have corrected the phrasing in the results section **(page 12, line 220)**.

Comment 13: Line 244 (Results): "We observed a significant immediate drop in quarterly persistence rates in Q2 2020..." – The ITS model likely measures the change starting in Q2 2020 (the intervention quarter), but the description could be clearer regarding the comparison (e.g., vs. the expected rate based on the pre-pandemic trend).

Response: We thank the reviewer for this helpful suggestion. We agree that our original phrasing could have been clearer in describing the Interrupted Time Series (ITS) model. To clarify this point, we have revised the sentence **(page 13, line 238)**.

Comment 14: Line 248-250 (Results, Figure caption): "of the pandemic. There was a significant drop in persistence rates in PY-1 q1 (March 15 to June 14, 2020) compared to the pre-pandemic period (coefficient: -24.99, p = 0.001), reflecting a level change at the onset of the pandemic." – This block of text appears to be a duplicate or misplaced description that should have been deleted during revision, as similar text exists earlier (Lines 242-244).

Response: We appreciate the reviewer's attention to detail. We have double-checked the current manuscript and confirmed that this description appears only once in the Results section, where we report the Interrupted Time Series (ITS) model results. It does not appear duplicated or misplaced. No changes were necessary in response to this comment.

Comment 15: Line 273 (Discussion): "among nonvalvular atrial fibrillation (NVAF) patients" – The abbreviation NVAF is introduced but not used again; AF is used elsewhere.

Response: Thank you for noting this. We agree that the terminology was inconsistent. To improve clarity and consistency, we have removed the abbreviation “NVAF” and now refer to the study population uniformly as patients with atrial fibrillation (AF) throughout the manuscript **(page 15, line 261)**.

Comment 16: Line 284-285 (Discussion): "This represents a much steeper drop than typically reported in what we might expect from the literature." – Awkward and grammatically incorrect phrasing.

Response: Thank you for your comment. The phrasing in this sentence has already been revised for clarity in the previous version of the manuscript.

Comment 17: Line 286 (Discussion): "reported an annual 12.5% annual adherence decline" – Repetition of "annual".

Response: We thank the reviewer for noting this wording issue. The duplication of the word “annual” appeared in an previous tracked-changes version. In the revised manuscript, this has already been corrected **(page 15, lines 272-274)**.

Comment 18: Line 287 (Discussion): "our pre-pandemic decline (21.6percentagepoint drop)" – Missing spaces: "21.6 percentage point".

Response: Thank you for pointing out this typographical issue. We have corrected the spacing and hyphenation in the manuscript **(page 15, line 274)**.

Comment 19: Line 295-298 (Discussion): "The sharper drop in our study at the onset of the pandemic in our study likely reflects may be explained by the significant major barriers substantial challenges faced by patients faced in continuing maintaining their OAC therapies," – Contains multiple editing errors from tracked changes, making it nonsensical.

Response: Thank you for pointing this out. The sentence had indeed been affected by unresolved tracked changes in the previous version. No changes were necessary in response to this comment.

Comment 20: Line 303 (Discussion): "By contrast, Hernandez et al. reported..." – "By contrast" is not formatted as the beginning of a new sentence.

Response: Thank you for this comment. This issue was addressed in the previous revision of the manuscript. No changes were necessary in response to this comment.

Comment 21: Line 305-307 (Discussion): "This could be consistent with the dramatic change of OAC persistence at the initial phase of the pandemic, as depicted in our interrupted time series analysis. On the contrary, Hernandez et al. reported increased OAC possession—higher with NOACs—at the first three months of the pandemic based on claims data [15]." – This appears to be leftover text from a previous draft that contradicts the revised narrative and should have been deleted.

Response: Thank you for this careful reading. We reviewed the current version of the manuscript and confirm that the sentences beginning with "This could be consistent with the dramatic change of OAC persistence…" and "On the contrary, Hernandez et al. reported…" do not appear in the revised text. These phrases were indeed part of an earlier tracked-changes draft and were removed in the previous revision.

Comment 22: Line 320 (Discussion): "reinforcing the need for contextual interpretation when comparing studies, claims sources" – Missing period and space, creating a run-on error.

Response: Thank you for this comment. In the current revised manuscript, this sentence has already been corrected. No changes were necessary in response to this comment.

Comment 23: Line 321-336 (Discussion): A large block of text (from "capture dispensing..." to "...clinical context.") is a duplicate paragraph that repeats, in slightly different wording, the explanation given in the previous paragraph (Lines 309-320). This is a major editing error.

Response: Thank you for pointing this out. In the earlier tracked-changes version, there was indeed redundant text explaining the contrast with Hernandez et al. In the current revised manuscript, we have removed the duplicated material and retained a single, streamlined paragraph that contrasts our EHR-based findings with claims-based results. The Discussion now includes only one consolidated paragraph (beginning "However, not all studies have reported similar findings. For instance, Hernandez et al.…" and ending "…when comparing studies."), so the redundancy has been resolved. No changes were necessary in response to this comment.

Comment 24: Line 337 (Discussion): "Adherence can be described as the use of drugs according to the recommended dose and duration, with a medication possession ratio of ≥80% considered good adherence [29]." – Abrupt, simplistic topic sentence that interrupts the flow of the discussion.

Response: Thank you for this observation. In the previous version of the manuscript, we had a topic sentence defining adherence in general terms. In the revised manuscript, this sentence

has been removed and the surrounding paragraph has been streamlined to maintain the flow of the discussion. No changes were necessary in response to this comment.

Comment 25: Line 339 (Discussion): "In our cohort of persistent patients, of persistent patients," – Repetition.

Response: We thank the reviewer for pointing this out. The duplicated phrase ("of persistent patients") was present in an earlier tracked-changes version. In the revised manuscript, this sentence has been edited for clarity. No changes were necessary in response to this comment.

Comment 26: Line 351 (Discussion): "Such This level of poor adherence likely has devastating consequences," – Editing error ("Such This").

Response: We agree that this wording was problematic in the previous version. In the current manuscript, this phrase has been fully revised and is no longer present. No changes were necessary in response to this comment.

Comment 27: Line 352-354 (Discussion): "Even brief discontinuation periods as short as one week have been associated with mortality, regardless of the OAC used [30], underscoring the need for more stringent adherence benchmarks." – Editing error ("brief as"), double period at the end.

Response: We appreciate this suggestion. We have revised the sentence to remove the redundant phrasing and potential punctuation issue **(page 17, line 316)**.

Comment 28: Line 356-359 (Discussion): "Moreover, the threshold of 80% for adherence is somewhat arbitrary, with recent calls for clinicians to adopt higher thresholds, especially for NOACs [31]. In fact, adherence rates of less than 95% were associated with increased risk of all-cause death [4]." – This appears to be leftover text from a previous draft, as the same point is made more clearly in the newly added sentences preceding it (Lines 348-355).

Response: We thank the reviewer for this careful reading. In the previous tracked-changes version, there was indeed some redundancy in how we discussed the ≥80% adherence threshold and the rationale for considering higher cutoffs. In the revised manuscript, we have already consolidated this into a single paragraph in the previous revision. No changes were necessary in response to this comment.
