# Supplementary material for: The impact of the COVID-19 pandemic on oral anticoagulation adherence in patients with atrial fibrillation managed in primary care: Results from the PRIME Registry
Source: PLoS One. 2026 Mar 6;21(3):e0344020. doi: 10.1371/journal.pone.0344020 (PMC12965529; doi:10.1371/journal.pone.0344020)
Supplement: Attachment — Submitted filename: PLOS Review.docx [file pone.0344020.s001.docx]

**PONE-D-25-29422**

**Abstract**

The finding for NOACs is unclear ("higher during the pandemic year-2" - higher what?).

The chosen pre-pandemic persistence comparison (2018-19 vs. pandemic year-1) is misleading; the immediate pre-pandemic year (2019-20) is more relevant.

The persistence drop is stated as "from 49.9% in 2018–2019 to 8.2% in the pandemic year-1". However, "2018-2019" is the Pre-2 period, but the most relevant pre-pandemic comparison is the immediate pre-pandemic year (Pre-1: 28.3%). The chosen comparison exaggerates the drop. The comparison to Pre-1 (28.3% to 8.2%) is still stark and more accurate.

**Methods**

Incorrect study design classification; this is a cohort study, not a cross-sectional study.

The "Post-1" period (March 2022-2023) is mislabeled as "post-pandemic"; it was still during the pandemic.

Calculating adherence (PDC) only among persistent patients is a major methodological flaw that inflates adherence rates and limits comparability.

**Results**

Table 2 is confusing; the sub-categories of adherence do not clearly sum to the "Good adherence" total.

The results state "Adherence rates increased to 50.8% in PY-2 and 57.0% in Post-1". Given the drastic drop in persistence (to 4.1% and 2.6% in these periods), this "increase" is based on a tiny, potentially highly selected subset of patients.

Reporting an "increase" in adherence in later pandemic years (PY-2, Post-1) is misleading without the context of a catastrophically low persistence rate (<5%), making the sample size tiny and unrepresentative.

**Discussion**

The explanation for the contrast with Hernandez et al. is convoluted and unclear.

The rationale for why rural/high SDI patients had better outcomes is speculative and unsupported by data.

**Conclusions**

Major contradiction; text recommends interventions for "low social deprivation areas," but results show "high" SDI was associated with better outcomes.

**Figures**

Figures (Fig 2, 3a, 3b) are cited in the text but were not provided for review.
