# Supplementary material for: The impact of the COVID-19 pandemic on oral anticoagulation adherence in patients with atrial fibrillation managed in primary care: Results from the PRIME Registry
Source: PLoS One. 2026 Mar 6;21(3):e0344020. doi: 10.1371/journal.pone.0344020 (PMC12965529; doi:10.1371/journal.pone.0344020)
Supplement: Attachment — Submitted filename: Review File 1.docx [file pone.0344020.s003.docx]

**Review PONE-D-25-29422R1**

**Line 41-42 (Abstract):** "NOAC use was associated with lower persistence pre-pandemic-, but with higher persistence during the pandemic year-2 compared to warfarin, but higher during the pandemic year-2." – This sentence is garbled, contains a stray hyphen and comma, and is repetitive.

**Line 49 (Keywords):** "Warfarin, NOAC" – Inconsistent formatting; other keywords are separated by semicolons.

**Line 80 (Methods):** "retrospective cohort cross-sectional study" – Appears to be a tracked-changes error, showing both "cohort" and "cross-sectional".

**Line 128 (Methods):** "A patient was considered persistent if she has an active prescription..." – Tense inconsistency ("was considered" vs. "has"). Also, "she" is gendered language not used elsewhere.

**Line 135-136 (Methods):** "avoids the underestimation that can occur when early discontinuers who by definition have zero coverage after discontinuation are included in the denominator to avoid potential confounding of early discontinuer compliance [17]," – Incomplete sentence and redundant phrasing due to tracked changes.

**Line 199-200 (Methods, Ethics):** "as outlined under the Department of Health and Human Services (HHS) guidelines (45 CFR 46.104, category 4)." – Sentence fragment.

**Line 214-217 (Results):** "Persistence rates were highest during the Pre-2 period at 49.9% and lower declined in the following subsequent periods:" – Contains editing errors ("lower declined") and redundant words ("following subsequent").

**Line 218-220 (Results):** "Adherence rates also had a general downward trend, starting at 65.1% in Pre-2 and 64.1% in Pre-1 and falling to 48.6% in PY-1. Adherence rates–These rates reflect annual treatment anniversary persistence," – First sentence is incomplete. The dash after "rates" creates a fragment.

**Line 221-223 (Results):** "Adherence rates, calculated among persistent patients only, also declined overall. They started at 65.1% in Pre-2 and 64.1% in Pre-1, then dropped to 48.6% in PY-1. Adherence rates appeared to increase to 50.8% in PY-2 and 57.0% in post-1 though rates remained below pre-pandemic levels;" – This repeats the adherence rates from the previous, now-incomplete sentence (Lines 218-220), creating redundancy and confusion.

**Line 224 (Results):** "these estimates are based on a very small subset of persistent patients (4.1% and 2.6% of the sample, respectively), increased to 50.8% in PY-2 and 57.0% in post-1." – This is a run-on sentence that incorrectly appends a repeated fact to a parenthetical clause.

**Line 225 (Results):** "Poor adherence rates were most prevalent..." – Previously, the term used is "poor adherence" (noun), not "poor adherence rates".

**Line 227 (Results):** "The proportion of patients with high adherence (MPR >95%) ..." – Inconsistent metric; the study uses PDC, not MPR (Medication Possession Ratio).

**Line 244 (Results):** "We observed a significant immediate drop in quarterly persistence rates in Q2 2020..." – The ITS model likely measures the change starting in Q2 2020 (the intervention quarter), but the description could be clearer regarding the *comparison* (e.g., vs. the expected rate based on the pre-pandemic trend).

**Line 248-250 (Results, Figure caption):** "of the pandemic. There was a significant drop in persistence rates in PY-1 q1 (March 15 to June 14, 2020) compared to the pre-pandemic period (coefficient: -24.99, p = 0.001), reflecting a level change at the onset of the pandemic." – This block of text appears to be a duplicate or misplaced description that should have been deleted during revision, as similar text exists earlier (Lines 242-244).

**Line 273 (Discussion):** "among nonvalvular atrial fibrillation (NVAF) patients" – The abbreviation NVAF is introduced but not used again; AF is used elsewhere.

**Line 284-285 (Discussion):** "This represents a much steeper drop than typically reported in what we might expect from the literature." – Awkward and grammatically incorrect phrasing.

**Line 286 (Discussion):** "reported an annual 12.5% annual adherence decline" – Repetition of "annual".

**Line 287 (Discussion):** "our pre-pandemic decline (21.6percentagepoint drop)" – Missing spaces: "21.6 percentage point".

**Line 295-298 (Discussion):** "The sharper drop in our study at the onset of the pandemic in our study likely reflects may be explained by the significant major barriers substantial challenges faced by patients faced in continuing maintaining their OAC therapies," – Contains multiple editing errors from tracked changes, making it nonsensical.

**Line 303 (Discussion):** "By contrast, Hernandez et al. reported..." – "By contrast" is not formatted as the beginning of a new sentence.

**Line 305-307 (Discussion):** "This could be consistent with the dramatic change of OAC persistence at the initial phase of the pandemic, as depicted in our interrupted time series analysis. On the contrary, Hernandez et al. reported increased OAC possession—higher with NOACs—at the first three months of the pandemic based on claims data [15]." – This appears to be leftover text from a previous draft that contradicts the revised narrative and should have been deleted.

**Line 320 (Discussion):** "reinforcing the need for contextual interpretation when comparing studies, claims sources" – Missing period and space, creating a run-on error.

**Line 321-336 (Discussion):** A large block of text (from "capture dispensing..." to "...clinical context.") is a duplicate paragraph that repeats, in slightly different wording, the explanation given in the previous paragraph (Lines 309-320). This is a major editing error.

**Line 337 (Discussion):** "Adherence can be described as the use of drugs according to the recommended dose and duration, with a medication possession ratio of ≥80% considered good adherence [29]." – Abrupt, simplistic topic sentence that interrupts the flow of the discussion.

**Line 339 (Discussion):** "In our cohort of persistent patients, of persistent patients," – Repetition.

**Line 351 (Discussion):** "Such This level of poor adherence likely has devastating consequences," – Editing error ("Such This").

**Line 352-354 (Discussion):** "Even brief discontinuation periods as short as one week have been associated with mortality, regardless of the OAC used [30], underscoring the need for more stringent adherence benchmarks." – Editing error ("brief as"), double period at the end.

**Line 356-359 (Discussion):** "Moreover, the threshold of 80% for adherence is somewhat arbitrary, with recent calls for clinicians to adopt higher thresholds, especially for NOACs [31]. In fact, adherence rates of less than 95% were associated with increased risk of all-cause death [4]." – This appears to be leftover text from a previous draft, as the same point is made more clearly in the newly added sentences preceding it (Lines 348-355).
